# One Health approach at the heart of the French Committee for monitoring and anticipating health risks

**DOI:** 10.1038/s41467-023-43089-2

**Published:** 2023-11-21

**Authors:** Thierry Lefrançois, Bruno Lina, COVARS, Yvanie Caille, Fabrice Carrat, Simon Cauchemez, Julie Contenti, Annabel Degrées du Loû, Léa Druet-Faivre, Didier Fontenille, Patrick Giraudoux, Mélanie Heard, Xavier De Lamballerie, Roger Le Grand, François-Xavier Lescure, Véronique Loyer, Denis Malvy, Céline Offerle, Jocelyn Raude, Olivier Saint-Lary, Rémy Slama, Brigitte Autran

**Affiliations:** 1 CIRAD, DG, F-75116 Paris, France; 2 HCL, Institut des Agents Infectieux, CNR des virus à transmission respiratoire (dont la grippe), Hôpital de la Croix Rousse, Lyon, France; 3 Virpath, Centre International de Recherche en Infectiologie, Université de Lyon Inserm U1111, CNRS UMR 5308, ENS de Lyon, UCBL, Lyon, France; 4 Sorbonne-Université, Cimi-Paris, Inserm U1135, CNRS ERL8255, UPMC CR7 Paris, France; 5 Renaloo, Paris, France; 6 IPLESP, Sorbonne Université, Inserm, Hôpital Saint-Antoine, APHP, Paris, France; 7 Mathematical Modelling of Infectious Diseases Unit, Institut Pasteur, Université Paris Cité, UMR2000 CNRS Paris, France; 8 Department of Emergency Medicine, Nice University Hospital, Nice, France; 9 IRD, Ceped Université de Paris IRD Inserm, Paris, France; 10 Task officer in support of the COVARS, Département stratégie et partenariats, ANRS, Inserm, Paris, France; 11 IRD, Université de Montpellier, CNRS, UMR MIVEGEC, Montpellier, France; 12 Chrono-environment, University of Franche-Comté/CNRS, Besançon, France; 13 Health Policy Department, Terra Nova progressive Think tank, Paris, France; 14 Unité des Virus Émergents, UVE: Aix Marseille Univ, IRD 190, INSERM, 1207 Marseille, France; 15 Université Paris-Saclay, CEA, Inserm, Center for Immunology of Viral, Auto-immune, Hematological and Bacterial diseases (IMVA-HB/IDMIT), Fontenay-aux-Roses, France; 16 Service des maladies infectieuses et tropicales, hôpital Bichat – Claude Bernard, APHP & IAME Inserm UMRS 1137, Université Paris cité, Paris, France; 17 Alliance Nationale des Associations en Milieu de Santé, Fondation Claude Pompidou, Paris, France; 18 Department for Infectious and Tropical Diseases, University Hospital Centre of Bordeaux, Bordeaux, France; 19 Inserm, UMR 1219, IRD EMR 271, Bordeaux Population Health Research Centre, University of Bordeaux, Bordeaux, France; 20 Collectif TRT-5 CHV, Paris, France; 21 EHESP School of Public Health, Rennes, France; 22 Primary Care and Prevention Team, CESP, University Paris-Saclay, UVSQ, INSERM U1018, Villejuif, France; 23 Inserm, Public Health Thematic Institute and Team of Environmental Epidemiology Applied to Development and Respiratory Health, Université Grenoble Alpes, Paris and Grenoble, France

**Keywords:** Policy, Infectious diseases, Agriculture, Environmental sciences, Institutions

## Abstract

Following the COVID-19 pandemic, the French government established a committee for monitoring and anticipating health risks. In this Comment, the authors describe the One Health approach taken by the committee, and outline its aims, composition, and initial actions.

## Teaser

The French Committee for Monitoring and Anticipating Health Risks (COVARS) has been strengthening the One Health approach through its interdisciplinary and multi-sectoral composition, the emerging risks it addresses (Covid-19, Mpox, vector-borne diseases, avian influenza…), its holistic approach to risks and its position at the science-decision interface.

The COVID-19 pandemic demonstrated the need for a holistic health approach to anticipate, prepare, and manage health crises. In France, the COVID-19 scientific council, set up in March 2020, produced a prospective report^1,2^ on the lessons of the pandemic and the need for a One Health approach as defined by the One Health High-Level Expert Panel (OHHLEP) and the quadripartite (WHO, WOAH, FAO, and UNEP)^3,4^.

Accordingly, the French government decided, at the end of the state of health emergency in July 2022, to replace the scientific council by a *Committee for Monitoring and Anticipating Health Risks* (COVARS) under the auspices of both the health ministry and the higher education and research ministry. The main objectives were (i) to maintain an independent and transparent multidisciplinary scientific advisory committee in charge of providing the government with evidence-based recommendations on health risks, (ii) to have an integrated approach to health and therefore to include risks related to the environment and climate change, beyond infectious diseases, and (iii) to anticipate potential health crises in order to promote prevention and preparedness. The ministries clearly stated in their press release that a One Health approach was fully included in the COVARS missions^5^.

Here, we illustrate how this interdisciplinary and multi-sectoral committee at the interface between science and decision-making has contributed to enhancing and improving the understanding and implementation of the One Health approach in France. This is highlighted through the operational functioning of the committee and the content of its reports on several specific risks (COVID-19, Mpox, vector-borne diseases, highly pathogenic avian influenza (HPAI)).

## One Health in the multidisciplinary and multisectoral composition of the committee

The One Health approach was reflected in its multidisciplinary composition that includes 16 experts with field experience in human, animal, or environmental health sectors in different disciplines (epidemiology, modeling, virology, infectiology, general medicine and emergency, veterinary epidemiology and microbiology, entomology, ecology, immunology and vaccinology, sociology, population science, health policy, etc.). The full list of members with their disciplines is indicated in each published advice^6^. Importantly, several experts had a longstanding experience with One Health approaches to emerging, vector-borne, or environmental diseases.

In addition, three civil society representatives (patients’ associations and citizens’ representatives) provide an important non-expert opinion to the group. Their contributions complement the scientific expertise with experience and knowledge of health democracy, community health approaches, field management of emerging diseases, and of the most vulnerable groups. They participate in the early stages of the process to ensure that recommendations are balanced with real-life experiences and testimonies of relevant professions or charities. They participate in the evaluation of acceptability of COVARS recommendations by the civil society and of their sustainability.

The committee consults with many stakeholders from different sectors, including national and regional agencies and research organizations involved in human and veterinary surveillance, patient associations, breeder associations, non-governmental organizations, diagnostic and vaccine developers, and ministries.

Altogether, the COVARS build a broad and integrated view on each subject aligned with the new inclusive definition and characterization of One Health^3,4^.

## One Health in the priority risks

The COVARS responds to the French government’s requests or can seize itself of health risks identified as priorities. These are defined during specific seminars, updated regularly according to the sanitary situation, and discussed with the ministries. In its first year of operation, the COVARS published 10 reports or recommendations^6^:

- Seven, in direct response to Government requests (COVID-19, Mpox, and mRNA vaccines)
- Two, produced following an initial specific request on Dengue that the COVARS discussed and reformulated with the Ministries. COVARS first produced a general report on vector-borne diseases, followed by two more specific reports on Dengue, Zika, Chikungunya (*Aedes* sp. being the vector), and on West Nile and Usutu (*Culex* sp. being the vector)
- One, is entirely self-referred (HPAI). Several factors make HPAI a particularly significant One Health risk. First, it cannot be eradicated because of the immensity and diversity of its wildlife and domestic reservoirs. Second, the rapid transmission between birds and its severity in wild birds increases the risk of transmission to and between mammals, and ultimately to humans. Third, climate change is altering the migratory patterns of wild birds and possibly their resistance to viruses, thus favoring the emergence of new HPAI outbreaks.

The work on vector-borne diseases and HPAI shows how COVARS can prioritize complex and multi-sectoral issues based on its own expertise.

## One Health in the holistic analysis of the risks

### One Health recommendations as specific parts of some recommendations

In the Covid-19 report, COVARS recommended to monitor the circulation of variants in animals and assess the risks of reverse zoonosis through immune escape or recombination induced by circulation in animal species (mink, deer, and hamsters), which can be enhanced by intensive virus circulation in areas with close interaction between humans and wild animals (e.g. live markets in China).

Similarly, the Mpox report analyzed the risk of animal-to-human transmission, either in natural habitats with susceptible species (squirrels, marmots) having sporadic contact with humans, or in anthropic contexts with animals of unknown sensitivity being in close contact with humans (dormouse, pet rats, and mice, dogs, or zoo animals). The COVARS recommended monitoring infection in animals and not only in humans and suggested that eradication in Europe would be difficult if an animal reservoir was detected.

The report on the future of mRNA vaccines analyzed veterinary vaccines in development (avian influenza) and suggested the need to coordinate research and development processes between the animal and human sectors.

### One Health at the heart of the COVARS work and recommendation

The vector-borne diseases reports highlighted the need to develop a One Health approach for transmission studies, human and animal disease surveillance, vector surveillance and control, epidemiological analysis and modeling, and also to consider climate change and socioeconomic factors in risk assessment. The COVARS recommended a coordinated inter-ministerial and multi-sectoral commitment to the surveillance and control of vector-borne diseases and an evaluation of the effectiveness of vector control techniques and their impact on the environment and on human health.

Regarding HPAI, the COVARS recommended to reinforce and coordinate multidisciplinary and multi-sectoral measures aimed at tackling this risk: First, rapidly implement the poultry vaccination strategy based on the DIVA strategy (differentiating infected from vaccinated animals) as recommended by Anses and extend the recommendation of seasonal influenza vaccination to people at risk in contact with birds. Second, adapt prevention and farm management counter-measures by actively seeking alternatives to preventive slaughtering, by improving euthanasia conditions of farm animals, and by developing support programs for affected farmers. Third, expand and strengthen human and financial resources to intensify domestic and wild animal, human, and environmental surveillance. Fourth, facilitate the collaboration between veterinary and human medicine, and allow human respiratory samples to be collected either by self-sampling or by veterinarians with diagnosis to be carried out in the veterinary laboratories. Last, create a multi-disciplinary emergency unit that can be activated rapidly in the event of a crisis.

## One Health in the science-decision and science–society interactions

The COVARS also operates in close interaction with other ministries (agriculture, ecological transition, foreign affairs, and overseas territories) all being essential for a One Health inter-ministerial approach. More specifically, the COVARS interacts with the technical directorates of these ministries but also with the cabinets and the Ministers themselves under the prime Minister cabinet umbrella.

The COVARS has also established strong links with human and animal health agencies, with their expert groups, and with the main research organizations involved in One Health. Auditions of specialized expert groups or research teams feed its recommendations and conversely, its reports can recommend to set up new expert groups or research work.

These regular meetings with ministries, agencies, and research organizations foster nationwide collaborations. Such close interactions between experts and decision-makers also contribute to the appropriation of the One Health concept, and favor its practical implementation at the national level. It also helps to follow the actual impact of the COVARS recommendations.

The COVARS recommendations are published on the ministry’s websites, and disseminated through press conferences, TV or radio interviews, or in the written press, all contributing to the dissemination of the One Health concept in society and to the literacy of the population on this matter. This is to be pursued through participatory research, communication, and youth education.

The current composition of the committee already promotes bridges with the civil society and the inclusion of a social psychologist in addition to the population scientist should help foster empowerment and general population engagement.

## Discussion and perspectives

Much remains to be done to strengthen efficient and transparent interactions between scientists, stakeholders, citizens, and decision-makers on One Health issues, and to improve inter-ministerial cooperation, concrete actions, and societal engagement on the issue.

Several COVARS reports have identified general weaknesses in policy responses that should be addressed: administrative issues for One Health research and implementation, lack of long-term research funding, lack of links between health databases from different sources, work in silos, weakness of social sciences and primary care research on emerging diseases, difficulties in evaluating multidisciplinary researches, weak links between research and surveillance.

Given the growing evidence of the interrelations between emerging diseases and climate change^7^, and/or biodiversity loss^8^, the COVARS will address health, climate change, and biodiversity crises altogether. It recommends not only to promote this global and complex approach within research and surveillance, but also, for a long-term impact, to train health professionals and decision-makers, and enhance public education.

Obviously, the COVARS is not the only structure implementing or favoring One Health in France. Ministries, research organizations, health agencies, civil society, or regions are key to strengthen this approach.

Other countries have also recently strengthened the framework for implementing One Health. In the USA, a new office for pandemic preparedness and response has been established, with a broad integrated approach, and a One Health bill to prevent, detect, and respond to biological threats was introduced in late 2022. Senegal has chosen to appoint a One Health advisor to the government. Altogether, the national implementation of One Health is still very heterogeneous in terms of structure and impact, yet.

Promoting inter-sectoral collaboration at the territorial level to better monitor emerging diseases is also critical. In addition, bringing together all the stakeholders and the public at this scale should help defining a healthy territory, how to monitor it, and the measures to be implemented to maintain it harmoniously.

Scaling up at the international level is also essential. We need to build on and strengthen existing regional One Health networks, involving all countries with the same regional epidemiological characteristics and emerging risks, regardless of their resources.

The COVARS aims at interacting more with similar structures in Europe to establish guidelines for such committees^9^ by sharing recommendations, discuss on transboundary health issues, and favor interactions with international initiatives tackling pandemic risk like Prezode^10^. National and regional One Health strategies should be supported by the quadripartite, which can provide guidance for better implementation of One Health at the global level (One Health Joint Plan of Action^11^).

Efficient implementation of One Health interventions requires a methodology based on multisectoral and multidisciplinary collaborations, joint active participation and cooperation, shared investments and resources, planning, and monitoring to allow evaluation of the social and economic benefits of this approach^12^. The COVARS is currently working with the French ministries to implement a monitoring and evaluation strategy of its recommendations.
